# Endogenous α7 nAChR Agonist SLURP1 Facilitates *Escherichia coli* K1 Crossing the Blood-Brain Barrier

**DOI:** 10.3389/fimmu.2021.745854

**Published:** 2021-10-14

**Authors:** Xiaolong He, Lei Wang, Liqun Liu, Jie Gao, Beiguo Long, Feng Chi, Tongtong Hu, Yu Wan, Zelong Gong, Li Li, Peilin Zhen, Tiesong Zhang, Hong Cao, Sheng-He Huang

**Affiliations:** ^1^ Department of Microbiology, Guangdong Provincial Key Laboratory of Tropical Disease Research, School of Public Health, Southern Medical University, Guangzhou, China; ^2^ Department of Infectious Disease, Jiangmen Central Hospital, Affiliated Jiangmen Hospital of Sun Yat-Sen University, Jiangmen, China; ^3^ Saban Research Institute, University of Southern California, Children’s Hospital Los Angeles, Los Angeles, CA, United States; ^4^ Department of Pediatrics, The Second Xiangya Hospital, Central South University, Changsha, China; ^5^ Kunming Key Laboratory of Children Infection and Immunity, Yunnan Institute of Pediatrics, Kunming Children’s Hospital, Kunming, China

**Keywords:** SLURP1, *E. coli* K1 meningitis, blood-brain barrier, inflammation, α7 nAChR

## Abstract

Alpha 7 nicotinic acetylcholine receptor (α7 nAChR) is critical for the pathogenesis of *Escherichia coli* (*E. coli*) K1 meningitis, a severe central nervous system infection of the neonates. However, little is known about how *E. coli* K1 manipulates α7 nAChR signaling. Here, through employing immortalized cell lines, animal models, and human transcriptional analysis, we showed that *E. coli* K1 infection triggers releasing of secreted Ly6/Plaur domain containing 1 (SLURP1), an endogenous α7 nAChR ligand. Exogenous supplement of SLURP1, combined with SLURP1 knockdown or overexpression cell lines, showed that SLURP1 is required for *E. coli* K1 invasion and neutrophils migrating across the blood-brain barrier (BBB). Furthermore, we found that SLURP1 is required for *E. coli* K1-induced α7 nAChR activation. Finally, the promoting effects of SLURP1 on the pathogenesis of *E. coli* K1 meningitis was significantly abolished in the α7 nAChR knockout mice. These results reveal that *E. coli* K1 exploits SLURP1 to activate α7 nAChR and facilitate its pathogenesis, and blocking SLURP1-α7 nAChR interaction might represent a novel therapeutic strategy for *E. coli* K1 meningitis.

## Introduction

Despite the widespread use of antibiotics, sepsis and meningitis remain to be severe complications in premature neonates, leading to high morbidity and mortality (1–3). Thus, developing more targeted therapeutic methods for meningitis is urgently needed. *Escherichia coli* K1 (*E. coli* K1), an opportunistic pathogen in the gut, accounts for 17.7% of meningitis patients and causes a mortality rate of 40%–58% in developing countries (4, 5). Recent studies from our group have revealed that alpha 7 nicotinic acetylcholine receptor (α7 nAChR) mediated the key *E. coli* K1 meningitis pathogenesis by promoting bacteria migrating across the blood-brain barrier (BBB). α7 nAChR-deficient mice had a higher survival rate, lower pathogen counts, and less inflammatory responses in the brain tissues than the wild-type littermates upon *E. coli* K1 infection (6–8). Furthermore, we found memantine, an antagonist of α7 nAChR approved by the FDA for therapy of dementia, could ameliorate *E. coli* K1 meningitis very efficiently (9, 10). While these studies revealed the critical role of α7 nAChR in *E. coli* K1 meningitis, little is known about how *E. coli* K1 manipulates α7 nAChR to facilitate its translocation into the central nervous system (CNS).

As a ligand-gated ion channel, α7 nAChR is abundantly expressed throughout the brain, including brain microvascular endothelium and astrocytes, which are major components of the BBB (11–13). The α7 nAChR is activated by many endogenous and exogenous ligands. Nicotine, a notable exogenous α7 nAChR agonist derived from tobacco, has been reported to impair the BBB permeability and host-microbial defense *via* stimulation of α7 nAChR (14–18). Many researchers have demonstrated that children under 5 years who had exposure to tobacco smoke in the air suffered from a higher risk of meningitis (19, 20). In contrast to nicotine, secreted Ly6/Plaur domain containing 1 (SLURP1) is an endogenous α7 nAChR ligand. SLURP1 serves as a positive allosteric modulator to potentiate α7 nAChR activity effectively (21, 22). However, whether this endogenous α7 nAChR ligand could dampen host defense against *E. coli* K1 to promote its penetration of the BBB is unclear. Thus, the present study aimed to explore the role of SLURP1 in the pathological process of *E. coli* K1 meningitis.

## Materials and Methods

### Ethics Approval

The Medical Ethics Committee of Southern Medical University approved all of the animal experiments (Protocol number: L2018018). All the experiments on mice were done according to the corresponding guidelines. Every attempt was taken to minimize the number and suffering of mice used. We purchased neonatal C57BL/6 mice (8 days old) from the Animal Experimental Center of Southern Medical University. The α7 nAChR heterozygous (A7R^+/−^) mice with C57BL/6J background were obtained from the Jackson Laboratory (B6.129S7-Chrna7tm1Bay/J, Stock No: 003232, Bar Harbor, ME). Littermate A7R*^−/−^* and A7R^+/+^ (wild-type) mice were generated from the heterozygous for the experiment. All animals were specific pathogen free and were kept on a 12-h light/dark cycle and free to get food and water.

### Public Transcriptional Data and Analysis

Two transcriptional data of *E. coli* infection patients were retrieved from Gene Expression Omnibus database (GSE33341, GSE65088). The metadata and SLURP1 transcriptional levels, as measured by fragments per kilobase of exon per million reads mapped (FPKM), were directly extracted from the data sets.

### Chemicals and Reagents

The chemicals and reagents used in this study were obtained as follows: Sigma-Aldrich (St. Louis, MO, USA) for bull serum albumin (BSA), Evans blue, Triton X-100, Tween-20, 4′,6-diamidino-2-phenylindole (DAPI), isopropyl-β-d-thiogalactoside (IPTG), Coomassie brilliant blue G 250, rifampin, kanamycin, gentamicin, and methyllycaconitine citrate (MLA); Thermo Fisher Scientific (Waltham, MA, USA) for fluorescent α-bungarotoxin conjugates; Gibco (Thermo Fisher Scientific, Inc., Waltham, MA, USA) for penicillin G, streptomycin, glutamine, and pyruvate; Abcam (Cambridge, UK) for antibodies against SLURP1, α7 nAChR or β-actin; Proteintech (Proteintech Group, Chicago, IL, USA) for enzyme-linked immunosorbent assay (ELISA) kits. The rest reagents were purchased from Beyotime Institute of Biotechnology, Shanghai, China.

### Clone, Expression, and Purification of Recombinant SLURP1

In this study, the amino acid sequence of SLURP1 we used to construct recombinant was described by previously (23), which is secreted by N-terminal signal cleavage (23-103 aa, as showed in **Supplementary Figure S1A**). Total RNA was extracted and used to amplify the SLURP1 cDNA, using primers containing 5′ *BamH*I and 3′*Not* I restriction sites at their termini. The primers used in the study were: sense, 5′-CGGGATCCCTCAAGTGCTACACCTGCAA-3′, and antisense, 5′ TTGCGGCCGCTCAGAGTTCCGAGTTGCAGA-3′. The cDNA was ligated into *BamH*I- and *Not*I- digested pET-28a, and transformed into *E. coli* BL21(DE3). Bacteria was added into the LB broth (1:100, containing 50 μg/ml kanamycin) and incubated at 37°C for 3–4 h (OD≈1). Afterward, IPTG was added at the final concentration of 0.1 mM and incubated at 30°C for 8 h for induction of protein expression. The protein was expressed as inclusion body form and purified using His-Tagged Protein Purification Kit (KangWeiShiJi Inc, Beijing, China) at denatured condition according to the manufacturer’s instructions. Purified SLURP1 was refolded in a series of gradient solutions of urea containing 50 mM Tris-HCl (pH 7.0), 0.5 M l-arginine, 4 mM glutathione, 1 mM glutathione disulfide, and 20% glycerin. The potential endotoxin was removed by passing through a Detoxi-Gel Endotoxin Removing Gel (Pierce Biotechnology, Rockford, IL, USA).

### Bacterial Strains, Cell Lines, Invasion, and PMN Transmigration Assay

*E. coli* K1 strain RS218 (O18:K1:H7) was isolated from the cerebrospinal fluid (CSF) of a meningitis neonate and showed rifampicin-resistant property (8, 24). The brain heart infusion broth was used to culture *E. coli* K1 at 37°C for 14 h, with supplementation of rifampin (100 μg/ml). The immortalized human brain microvascular endothelial cells (HBMEC) were isolated and cultured as described previously (8, 24, 25). RPMI 1640 medium (Gibco; Thermo Fisher Scientific, Inc., Waltham, MA, USA) was used as basic medium, with the following supplementations: 10% fetal bovine serum from Gibco (Thermo Fisher Scientific, Inc., Waltham, MA, USA), 50 U/ml penicillin G, 50 μg/ml streptomycin, 2 mM glutamine, and 1 mM pyruvate according to previous studies (8, 25).

For invasion assays, HBMEC were cultured in 24-well plates and incubated with SLUPR1 (0.1–2 μg/ml) for 2 h, followed by infected with *E. coli* K1 [1 × 10^7^ colony-forming unit (CFU)] for another 2 h. To kill the extracellular bacteria, the HBMEC was washed twice with sterile PBS and incubated with RPMI 1640 medium containing gentamicin (100 μg/ml) for 1 h. Then, the HBMEC were washed again and lysed using sterile water. Internalized bacteria were counted by plating the cell lysates on Luria-Bertani broth agar (containing 100 μg/ml rifampicin).

Polymorphonuclear leukocytes (PMN) transmigration experiments were carried out as previously (8, 14, 25). HBMEC monolayers on transwell filters (3 μm pore size, 6.5 mm diameter, Corning, product number 3415) were monitored by measuring trans-endothelial electrical resistance (TEER) changes across the endothelial cell monolayer using an End Ohm epithelial voltohmeter (World Precision Instruments, Sarasota, FL, USA). To exclude the possibility that the PMN migration elicited was due to destruction of HBMEC monolayer, the integrity of the monolayer was inspected by TEER and microscopy before the start of the PMN transmigration assay. Transwell filters with or without supplementation of SLUPR1 (0.1–2 μg/ml) were employed to culture fully confluent HBMEC monolayers for 2 h. Then *E. coli* K1 (1 × 10^5^ CFU/ml) was added to the bottom of the Transwell filters and infected for another 2 h. Then PMNs were applied to the upper compartment at a concentration of 1 × 10^6^ cells. The Transwell filters system was kept at 37°C, 5% CO_2_. After incubated for 4 h, the Transwell filters were removed and migrated PMNs in the bottom of 24-well plates were harvested and counted in a blinded manner.

### Knockdown and Overexpression of SLURP1

SLURP1 expression was knockdown using RNA interference. In brief, predesigned siRNA specific for SLURP1 and nontargeting scrambled siRNA (control) were obtained from Santa Cruz Biotechnology (CA, USA). The Lipofectamine™3000 transfection reagents (Invitrogen, USA) were mixed with the siRNA solutions, applied to the HBMEC monolayers, and maintained at 37°C, 5% CO_2_ for 24 h. The transfected HBMECs were used for invasion and PMN transmigration assays as described above.

For overexpression of SLURP1, the full-length cDNA of SLURP1 was cloned into a pcDNA3.1(+) vector to construct the pcDNA3.1-SLURP1 expression plasmid. The control vector or pcDNA3.1-SLURP1 plasmid was transfected into HBMEC for 24 h using Lipofectamine™3000. The transfected HBMECs were used for invasion and PMN transmigration experiments as described above.

For the transfection in the Transwell filters, HBMECs were seeded onto Transwell filters and grown to confluency, then transfected with siRNA or SLURP1 expression vector as mentioned above. In order to ensure the barrier function remains comparable between different groups, HBMEC transfected with nontargeting scrambled siRNA or control vector were served as the scrambled control.

### Mouse Model of *E. coli* K1 Meningitis

From postnatal days 8 to 10, neonatal C57BL/6 mice were intraperitoneally injected with SLURP1 or BSA daily at a dose of 0–100 mg/kg body weight. To establish an *E. coli* K1 meningitis model, mice were injected with *E. coli* K1 (1 × 10^6^ CFU, in 20 μl PBS) intraperitoneally at day 10. Control mice were given 20 μl PBS using the same route of injection. After infection for 18 h, the blood samples were collected and plated on Luria-Bertani agar (containing 100 μg/ml rifampicin) plates. Puncture through cisterna magna were carried out to collect CSF samples, followed by inoculating into the Luria-Bertani agar plates (containing 100 μg/ml rifampicin). Mice were perfused with 30 ml sterile PBS by the intracardiac route. Then brain tissues were harvested under aseptic conditions and homogenized in saline. Serial tenfold dilutions of brain homogenates were carried out and plated on Luria-Bertani agar (containing 100 µg/ml rifampicin) for counting. CSF samples were stained with a FITC-Ly-6G (Gr-1) (ProteinTech Group, Chicago, IL, USA) antibody and counted under fluorescence microscopy for PMN counting. For Evans blue assay, mice were injected intraperitoneally with Evans blue at a concentration of 40 mg/kg body weight 3 h before sacrificing. After intracardiac perfused with 30 ml PBS, the brain tissues were harvested and immersed in formamide. The OD620 of the supernatant was measured using a spectrophotometry.

### Immunohistochemical Staining

Brain tissues were kept in formalin and transported to histological examination. After cutting into 3 µm sections, the tissues were hematoxylin-eosin (H&E) stained to assess tissue inflammation and damage. For the immunohistochemical staining, xylene was first used to dewax paraffin sections for 10 min, followed by gradient alcohol to dehydrated and rinsing in distilled water. Then the sections were heated in citrate buffer solution at 100°C for 40 min to retrieve the antigen. Hydrogen peroxide/methanol (30%) was used to stop endogenous peroxidase activity (45 min at 25°C). One percent BSA was used to block tissue sections and then incubated with an antibody specific for rabbit anti-SLURP1 (1:200, Abcam) at 4°C for 12 h. After repetitive washing by PBS, the sections were incubated with peroxidase-conjugated antirabbit antibodies, followed by visualization using 3,3-diaminobenzidine with hematoxylin counterstain. Immunostaining was quantified by ImageJ software.

For the immunofluorescence staining, the deparaffinization and antigen retrieval of sections were done as described above. Three percent normal goat serum and 0.1% Triton X-100 were used to block sections. After washing, sections were incubated with antibody specific for SLURP1 (1:200, Abcam) and a7 nAChR (1:200, Abcam) at 4°C for 16 h. After washing and incubating with appropriate secondary antibodies and DAPI, the sections were observed using fluorescence microscopy. NIH image analysis software (ImageJ) was employed to quantify the results of immunofluorescence staining.

### Fluorescent α-Bungarotoxin Binding

HBMEC cells cultured in 24-well tissue were treated with BSA (2 μg/ml), SLURP1 (2 μg/ml), *E. coli* K1 (1 × 10^5^ CFU) or *E. coli* K1 (1 × 10^5^ CFU) + anti-SLURP1 antibody (1 μg) at 37°C, 5% CO_2_ for 2 h. The SLURP1 antibody was added simultaneously with *E. coli* K1. After general washing, 4% paraformaldehyde were used to fix cells for 10 min and 5% BSA was employed to block cells for 30 min. Alexa Fluor 488-conjugated α-bungarotoxin was then added at a concentration of 1 μg/ml and kept for 6 h at room temperature. Followed by washing, the sections were observed using fluorescence microscopy. The relative fluorescence intensity was determined using the ImageJ software (NIH). Briefly, the Spot Enhancing Filter 2D plugin was used to amplify signals from the cells, and then threshold settings were used to specifically select the fluorescent regions. The selected regions were overlaid on the original images and analyzed for mean fluorescence intensity of the area.

### Immunoblot Analysis

The culture supernatants of HBMEC infected with or without *E. coli* K1 were concentrated using ultrafiltration for immunoblot analysis. Cell lysates were prepared in RIPA buffer. SDS-polyacrylamide gel was used to separated protein (20–30 μg), followed by transferring onto polyvinylidene difluoride membranes (Millipore). Five percent of skim milk was used to block membranes for 1 h. Membranes were then incubated with rabbit-anti-SLURP1 antibody (1:1,000, Abcam) at 4°C overnight. β-Actin (1:20,000) or Coomassie staining of total proteins was employed as an internal control. SLURP1 expression was detected using goat antirabbit IgG antibody conjugated with horseradish peroxidase and enhanced chemiluminescence reagent kit.

### ELISA

The proinflammatory cytokines TNF-α (ab208348, Abcam), MMP-9 (ab253227, Abcam), and ICAM-1 (ab100688, Abcam) from homogenized brain extracts were evaluated using ELISA kits according to the manufacturer’s instructions.

### Statistical Analysis

Data are shown in mean ± standard error. All the analyses in this study were done by SPSS (v25.0). Group differences between two groups were analyzed using the Student’s *t*-test. Group differences between three or more groups were analyzed using the one-way ANOVA followed by Bonferroni *post-hoc* test. Survival rates comparations were analyzed with log-rank test. Two-side *p*-value less than 0.05 was considered significant and is represented as *p < 0.05, **p < 0.01, and **p < 0.001.

## Results

### *E. coli* K1 Infection Induces SLURP1 Secretion in Cell Lines, Murine Model, and Humans

**Figure 1:**
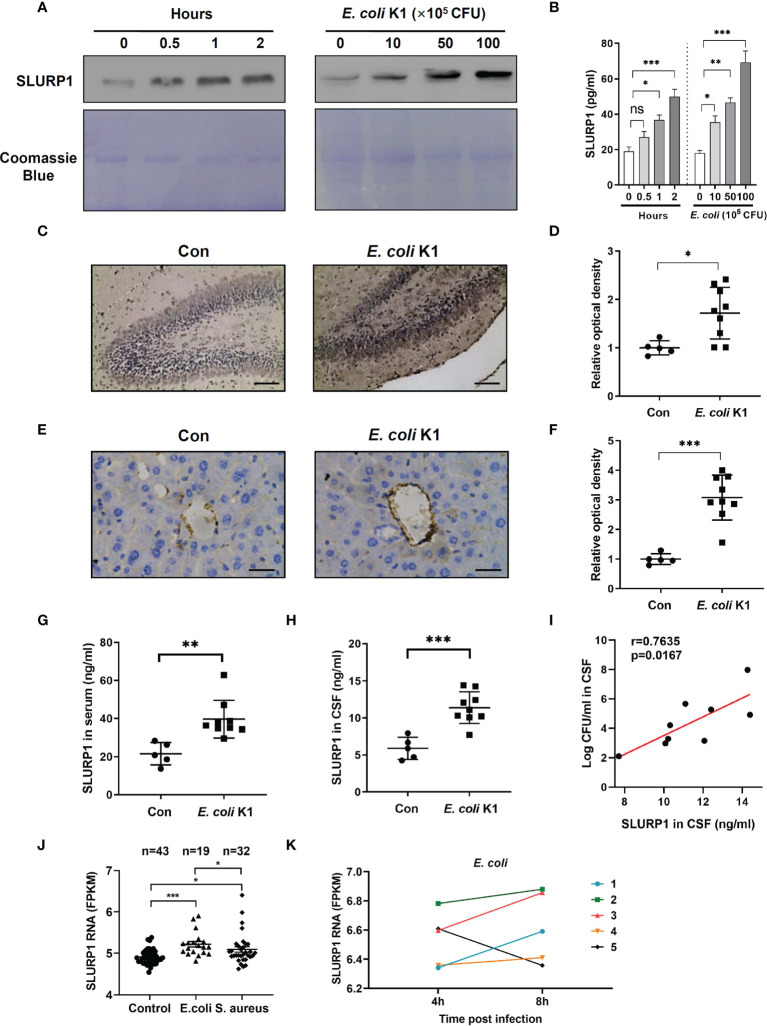
*E coli* K1 enhances SLURP1 secretion. **(A, B)** Immunoblot analysis **(A)** and ELISA **(B)** showed the SLURP1 release in the culture supernatant of HBMEC infected with *E coli* K1 (5 × 10^6^ CFU) at different time intervals (0–2 h, left panel), or infected with various doses of *E coli* K1 (0–1 × 10^7^ CFU) for 2 h (right). **(C)** Immunohistochemical staining of SLURP1 in the hippocampus sections of mice infected with or without *E coli* K1, scale bar = 200 μm. **(D)** The semiquantitative results of immunohistochemical staining of SLURP1, shown as fold change relative to control. **(E)** Immunohistochemical staining of SLURP1 in the cortex sections from mice challenged with or without *E coli* K1, scale bar = 40 μm. **(F)** Semiquantitative analysis of immunohistochemical staining of SLURP1, shown as fold change relative to control. **(G, H)** Quantification of SLURP1 secretion in the serum **(G)** or CSF **(H)** of mice infected with or without *E coli* K1. **(I)** Correlation analysis of SLURP1 levels and *E coli* K1 counts in the CSF. **(J)** Relative SLURP1 transcription levels (FPKM) among patients with *E coli* or *S. aureus* sepsis. **(K)** Change of relative SLURP1 transcription levels (FPKM) of five patients in 4- or 8-h post-*E. coli* infection. FPKM, fragments per kilobase of exon per million reads mapped. Data are presented as mean ± SEM. The immunoblots and immunohistochemical results are representative of two independent experiments **(A, C, E)**. The data are displayed as the mean ± SEM from two independent experiments **(B, D, F, G–I)**. *p < 0.05; **p < 0.01; ***p < 0.001 by one-way ANOVA followed by Bonferroni *post-hoc* test **(B, J)** and Student’s *t*-test **(D, F–H)**. Correlation analysis was performed using Pearson and Spearman correlation tests. ns, not significant.

Secretion of SLURP1 in the culture supernatants of *E. coli* K1-infected HBMEC were analyzed using immunoblot assay and ELISA. We found *E. coli* K1 infection enhanced the SLURP1 secretion in both time- and dose-dependent manner compared with uninfected HBMECs (**Figures 1A, B**). To confirm these results *in vivo*, we assessed the SLURP1 expression in brain sections of neonatal C57BL/6 mice infected with or without *E. coli* K1 by immunohistochemical detection. As shown in **Figures 1C, D**, SLURP1 protein expression from hippocampus areas was significantly increased in mice infected with *E. coli* K1 compared with that of control. Notably, we also found a lot of SLURP1 was specially gathered around the blood vessels in the cortex sections of mice infected with *E. coli* K1 (**Figures 1E, F**). ELISA showed that mice infected with *E. coli* K1 showed a higher concentration of SLURP1 both in the serum and CSF than that of control (**Figures 1G, H**). Pearson correlation analysis indicated that the concentrations of SLURP1 were positively correlated with *E. coli* K1 counts in the CSF (**Figure 1I**, *r* = 0.7635, *p* = 0.0167).

To identify if *E. coli* infection can upregulate SLURP1 transcription in humans, we analyzed one public human data set encompassed sepsis caused by *E. coli* or *Staphylococcus aureus* (*S. aureus*), and found that SLURP1 transcription level was higher in *E. coli*-infected patients, as compared with healthy controls or *S. aureus-*infected patients (**Figure 1J**). Furthermore, SLURP1 transcription levels get higher at 8 h postinfection than 4 h postinfection of *E. coli* (**Figure 1K**). Together, these results revealed that *E. coli* infection could induce SLURP1 secretion.

### SLURP1 Promotes *E. coli* K1 Invasion and PMN Transmigration Across the BBB *In Vitro*

**Figure 2:**
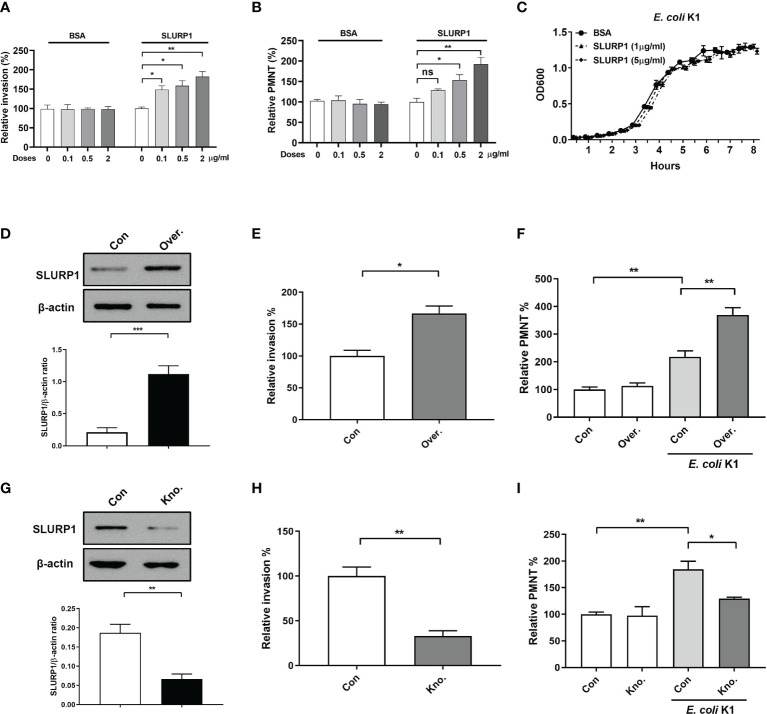
The effect of SLURP1 on *E coli* K1 penetration and PMN transmigration across the BBB *in vitro*. **(A)** The invasion of *E coli* K1 into HBMEC which pretreated with indicated doses of BSA or SLURP1. Data are presented as percent of the control values. **(B)** Transwell-cultured HBMEC monolayers were pre treatment with indicated doses of BSA or SLURP1 for 2 h, followed by incubated with *E coli* K1 in the bottom and PMN in the top of filter successively. PMN in the bottom of filter were harvested and counted. **(C)** The growth curve of *E coli* K1 in medium containing indicated doses of SLURP1. **(D)** The upregulation effect of SLURP1 in HBMEC which had been transfected with pCNDA3.1+-SLURP1. **(E)** The invasion of *E coli* K1 into HBMEC which had been transfected with pCNDA3.1+-SLURP1. Data are presented as percent of the control values. **(F)** Effect of SLURP1 upregulation on *E coli* K1-induced PMN transmigration across the HBMEC monolayers. Data are presented as percent of the control values. **(G)** The knockdown effect of SLURP1 in HBMEC transfected with siRNA. **(H)** The invasion of *E coli* K1 into HBMEC which had been transfected with siRNA. Data are presented as percent of the control values. **(I)** Effect of SLURP1 knockdown on *E coli* K1-induced PMN transmigration across the HBMEC monolayers. Data are presented as percent of the control values. Over., overexpression; Kno., knockdown. The immunoblots results are representative of three independent experiments **(D, G)**. The data are displayed as the mean ± SEM from three independent experiments **(A–I)**. *p < 0.05; **p < 0.01 by one-way ANOVA followed by Bonferroni *post-hoc* test **(A, B, F, I)** and Student’s *t*-test **(D, E, G, H)**. ns, not significant.

The pathogenesis of *E. coli* K1 meningitis required two key events: invasion of the brain microvascular endothelial cells by the bacteria and PMN transmigration across the BBB (8), we therefore next employed immortalized HBMEC monolayers to determine whether exogenous supplement of SLURP1 could promote *E. coli* K1 penetrating the endothelial cells, as well as support PMN transmigration across the BBB *in vitro*. SLURP1 was obtained recombinantly with a His-tag as described in the ***Material and Methods*** section. The amino acid sequence is shown in **Supplementary Figure S1A**. The results of double-digestion analysis, protein purity, and immunoblot detection, DNA sequencing are shown in **Supplementary Figures S1B, C** and **Supplementary Material 1**, respectively. As shown in **Figures 2A, B**, supplement with SLURP1 promoted *E. coli* K1 penetrating the endothelial cells, accompanied by enhancing *E. coli* K1-induced PMN transmigration across the HBMEC monolayers in a dose- and time-dependent manner. To explore the possibility that SLURP1 may promote *E. coli* K1 growth, we compared its growth on brain heart infusion broth in presence or absence of SLURP1. The result showed that SLURP1 has no obvious influence on *E. coli* K1 growth (**Figure 2C**), suggesting that the promotive effects of SLURP1 on *E. coli* K1 infection is not through promoting the growth of the pathogen. In order to further confirm the promotive role of SLURP1, we generated SLURP1 overexpression or knockdown HBMEC. **Figures 2D, G** showed the effects of SLUPR1 overexpression or knockdown, respectively. We found overexpression/knockdown of SLUPR1 significantly increased/decreased *E. coli* K1 penetrating the endothelial cells and PMN transmigration across the HBMEC monolayers, respectively (**Figures 2E, F, H, I**). Taken together, these results indicate that SLURP1 promotes *E. coli* K1 invasion of the endothelial cells, as well as enhances PMN transmigration across the BBB.

### SLURP1 Promotes the Pathogenesis Process of *E. coli* K1 Meningitis *In Vivo*

**Figure 3:**
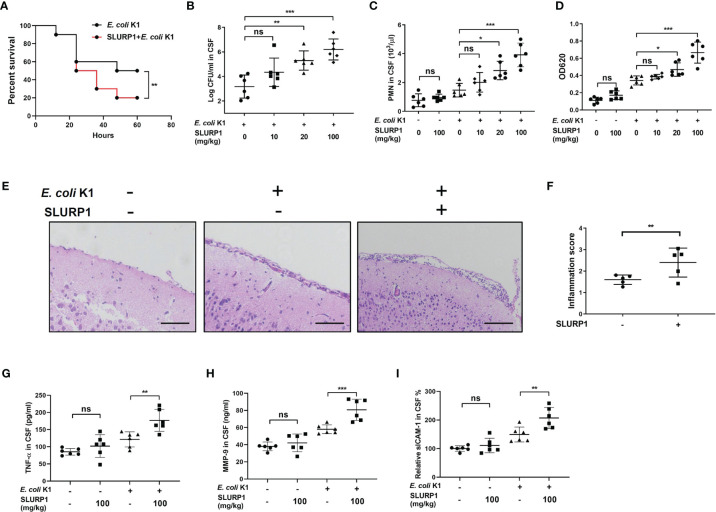
SLURP1 promotes the pathogenesis of *E coli* K1 meningitis in mice model. **(A)** Survival curve of C57BL/6 mice treated with SLURP1 (100 mg/kg body weight) + *E. coli* K1 (1 × 10^6^) or treated with only *E coli* K1 (1 × 10^6^). SLURP1 was intraperitoneally injected 2 days before *E coli* K1 challenge. *n* = 10 per group. **(B, C)** Pathogen **(B)** and PMN **(C)** counts in the CSF of *E coli* K1-infected mice pretreated with BSA or indicated doses of SLURP1. **(D)** The OD620 values of Evans blue extracted from the brain of *E coli* K1-infected mice pretreated with BSA or indicated doses of SLURP1. **(E)** Representative H&E staining of the cortex sections, scale bar = 200 μm; and **(F)** meningeal inflammation score. **(G–I)** The cytokines levels in the CSF were analyzed by ELISA: TNF-α **(G)**, MMP-9 **(H)**, and ICAM-1 **(I)**. The H&E staining are representative of two independent experiments **(E)**. Data are presented as mean ± SEM from two independent experiments. Each dot indicates an individual mouse (*n* = 5). *p < 0.05; **p < 0.01; ***p < 0.001 by log-rank test **(A)**, Student’s *t*-test **(F)**, and one-way ANOVA followed by Bonferroni *post-hoc* test **(B–D, G–I)**. ns, not significant.

In order to confirm the biological significance of the *in vitro* findings described above, we further tested the effect of SLURP1 on *E. coli* K1 meningitis pathogenesis in the murine model. Neonatal mice were intraperitoneally administered with BSA or SLURP1 two consecutive days prior to *E. coli* K1 challenge. The survival rate, pathogen counts in CSF, PMN transmigration, and brain damage were detected as described in ***Materials and Methods***. The results showed that only 20% of the SLURP1-pretreated mice infected with *E. coli* K1 survived within 60 h postinfection (**Figure 3A**), while the survival rate for *E. coli* K1-infected mice without SLURP1 supplementation reached almost 50%. Furthermore, we found administration with SLURP1 was able to markedly increase pathogen and PMN counts in the CSF (**Figures 3B, C**). Evans blue assay showed that SLURP1-pretreated mice have more severe BBB damage than the control group (**Figure 3D**). Notably, we found SLURP1 has no influence on the BBB integrity of uninfected mice. H&E staining of brain sections indicated that supplement with SLURP1 dramatically promotes neutrophil infiltration into the meninges and meningeal inflammation (**Figures 3E, F**). Additionally, we found exogenous supplement of SLURP1 could robustly enhance the levels of proinflammatory cytokines in brain homogenates (**Figures 3G–I**). Above all, these results suggested that SLURP1 promotes the pathogenesis process of *E. coli* K1 meningitis.

### *E. coli* K1-Induced SLURP1 Activates α7 nAChR

**Figure 4:**
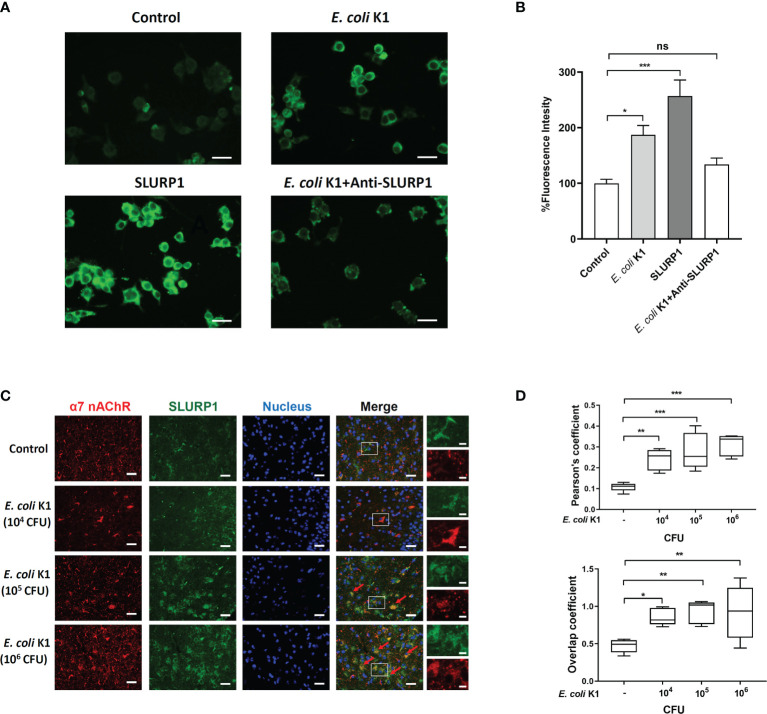
*E coli* K1-induced SLURP1 activates α7 nAChR. **(A)** The Alexa Fluor 488-conjugated α-bungarotoxin binding to HBMEC was detected upon treatment with BSA (control, 2 μg/ml), *E coli* K1 (1 × 10^5^ CFU), SLURP1(2 μg/ml) or *E coli* K1 (1 × 10^5^ CFU) + SLURP1 antibody (1 μg). SLURP1 antibody was added simultaneously with *E coli* K1. Scale bars = 40 μm. **(B)** Fluorescence intensity of Alexa Fluor 488-conjugated α-bungarotoxin binding to HBMEC. Data are presented as percent of the control values. **(C)** The cortex sections of neonatal mice infected with indicated doses of *E coli* K1 were permeabilized and immunofluorescence stained with either α7 nAChR and SLUPR1 antibodies. Nuclei were stained by DAPI. Arrows show the colonization of α7 nAChR (red) and SLUPR1 (green). Scale bars = 50 μm (left) or 10 μm (right). **(D)** Quantification of the Pearson’s correlation coefficient (upper panel) and overlap coefficient (bottom) for colonization of α7 nAChR and SLURP1. Data are presented as mean ± SEM from three independent experiments. *p < 0.05; **p < 0.01; ***p < 0.001 by one-way ANOVA followed by Bonferroni *post-hoc* test **(B, D)**. ns, not significant.

We further confirmed if *E. coli* K1-induced SLURP1 activates α7 nAChR. Firstly, a fluorescent α-bungarotoxin (α-bgtx) binding assay was performed to detect the activity of α7 nAChR. HBMEC were cultured in 24-well plates, followed by incubating with SLURP1 or *E. coli* K1 for 2 h. Afterward, fluorescently labeled α-bgtx incubation was carried out to test α7 nAChR activity. As shown in **Figures 4A, B**, SLURP1 or *E. coli* K1-treated HBMEC showed brighter green fluorescence than control, indicating an increase in α7 nAChR activity. Notably, when added with SLURP1 antibody, the promotive effect of *E. coli* K1 on α7 nAChR activity was blocked, indicating the *E. coli* K1-induced SLURP1 was responsible for the α7 nAChR activation. To further confirm whether SLURP1 is directly linked to α7 nAChR activation, we analyzed the colocalization of fluorescently labeled SLURP1 with α7 nAChRs by immunofluorescence staining. The cortex sections of *E. coli* K1-treated mice showed colocalization of SLURP1 (green) and α7 nAChRs (red) (**Figure 4C**). What is more, with increasing doses of *E. coli* K1 challenge, the Pearson’s coefficient and overlap coefficient of colocalization also increased (**Figure 4D**). These results suggest that *E. coli* K1-induced SLURP1 is responsible for α7 nAChR activation.

### Inhibition of α7 nAChR Blocks the Promotive Effects of SLURP1 in the Pathogenesis of *E. coli* K1 Meningitis

**Figure 5:**
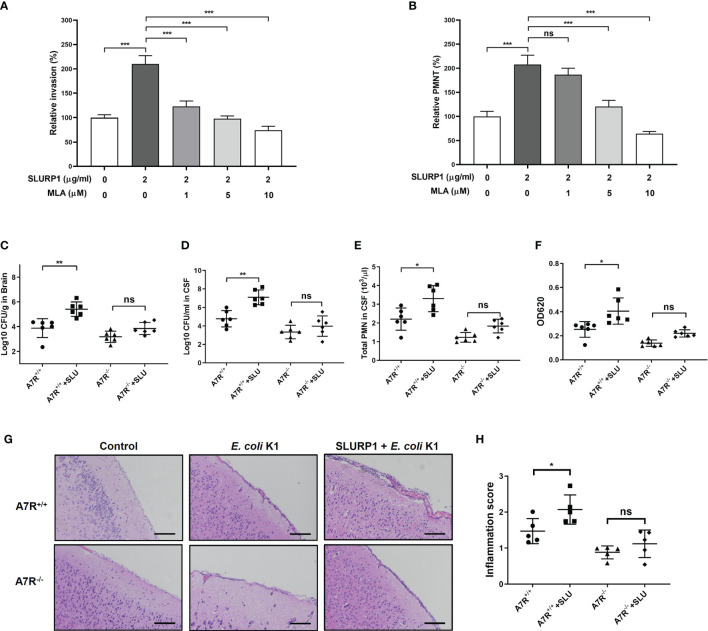
Effects of α7 nAChR knockout on SLURP1-enhanced *E coli* K1 entry into the CNS and PMN transmigration across the BBB. **(A)** The invasion of *E coli* K1 into HBMEC which pretreated with indicated doses of SLURP1 or SLURP1+α7 nAChR inhibitor MLA. Data are presented as percent of the control values. **(B)** *E coli* K1-induced PMN transmigration across the HBMEC monolayers which pretreated with indicated doses of SLURP1 or SLURP1+α7 nAChR inhibitor MLA. Data are presented as percent of the control values. **(C, D)** Pathogen counts in the brain tissues **(C)** or CSF **(D)** of *E coli* K1-infected wild type (A7R^+/+^) or α7 nAChR knockout (A7R^−/−^) mice pretreated with or without SLURP1 (100 mg/kg body weight). **(E)** PMN counts in the CSF of *E coli* K1-infected A7R^+/+^ or A7R^−/−^ mice pretreated with or without SLURP1 (100 mg/kg body weight). **(F)** Measurement of the BBB permeability by Evans blue assay in *E coli* K1-infected A7R^+/+^ or A7R^−/−^ mice administrated with or without SLURP1 (100 mg/kg body weight). **(G)** Representative H&E staining of the brain cortex sections, scale bar, 200 μm, and **(H)** meningeal inflammation score. The H&E staining are representative of two independent experiments **(G)**. Data are presented as mean ± SEM from two independent experiments. Each dot indicates an individual mouse. *p < 0.05; **p < 0.01; ***p < 0.001 by one-way ANOVA followed by Bonferroni *post-hoc* test **(A–F**, **H)**. ns, not significant.

Finally, we determined whether α7 nAChR is necessary for SLURP1-enhanced *E. coli* K1 meningitis. We first used the MLA, an α7 nAChR inhibitor, to explore the role of α7 nAChR on the function of SLURP1 *in vitro*. As shown in **Figures 5A, B**, MLA inhibited the promotive effects of SLURP1 in a dose-dependent manner, including attenuating *E. coli* K1 invasion and PMN transmigration. Furthermore, we used α7 nAChR knockout (A7R^−/−^) mice to confirm these findings *in vitro*. Wild-type (A7R^+/+^) and A7R^−/−^ mice were intraperitoneally injected with BSA or SLURP1 for 2 days, followed by challenge with *E. coli* K1 for 18 h. As shown in **Figures 5C**–**F**, SLURP1 treatment has enhanced the pathogen load, PMN transmigration and the BBB damage in the A7R^+/+^ mice, while all these promotive effects of SLURP1 were blocked in the A7R^−/−^ mice. H&E staining of the brain sections showed that neutrophil recruitment and meningeal inflammation of SLURP1-treated A7R^−/−^ mice was not significantly increased when compared with untreated A7R^−/−^ mice (**Figures 5G, H**). Above all, these results indicated that SLURP1 acts through α7 nAChR to enhance the pathogenesis process of *E. coli* K1 meningitis.

## Discussion

As an important cholinergic anti-inflammatory signaling, α7 nAChR has been intensively investigated in sterile inflammation over the last decades (26–30). However, there are few studies that focus on the role of α7 nAChR in the host immune response to microbial infection. A number of studies showed that activation of α7 nAChR impairs host defense to bacterial infections, indicating that the use of α7 nAChR ligands might not be a good strategy in treating infectious diseases (31–37). Ilona and coworkers have demonstrated that stimulates α7 nAChR promotes the development of *E. coli* peritonitis after intraperitoneal infection (37). Consistent with their study, our group recently revealed that α7 nAChR impaired the host defense against *E. coli* K1 infection in the CNS (7–9). In this report, we further found that *E. coli* K1 infection promotes SLURP1, an endogenous α7 nAChR ligand secretion, and supplement of SLURP1 could greatly facilitate *E. coli* K1 dissemination into the CNS. These findings expand our knowledge of the pathogenesis process of *E. coli* K1 infection and provide a new perspective on the establishment of the CNS infection.

SLURP1 is a secreted protein that has structural homology with three-finger snake α-neurotoxins, acts in a both autocrine and paracrine manner, to activate α7 nAChR and thus exert potent anti-inflammatory activity (38–43). Although the function of SLURP1 in modulated sterile inflammation has been extensively studied, its effects on the inflammation induced by microbial infection are largely unknown, especially on neuroinflammation. Taken into account that SLURP1 is highly expressed in the CNS, a study into its pathologist effects on the CNS infection constitutes a promising field for developing new therapeutic methods (44–46). To the best of our knowledge, this is the first study that reported that microbial infection could induce SLURP1 expression, and consequently stimulate α7 nAChR to establish infection. Whether the enhanced SLURP1 secretion is a universal phenomenon upon infection is very interesting and warrants further investigation.

How *E. coli* K1 promotes SLURP1 secretion needs to be addressed. Kruppel-like factor 4 (Klf4) is the first reported factor that regulates SLPRP1 expression (47, 48). Klf4 is a member of the Krüppel-like factor transcription factor family, which can stimulate microglial activation and induce neuroinflammation. As a zinc finger protein, Klf4 could effectively induce SLURP1 expression *via* binding to its promoter. Interestingly, two recent studies reported that Klf4 is robustly upregulated upon infection with pathogens like *E. coli* or *Streptococcus pneumoniae* (49, 50). Thus, we speculated that *E. coli* K1 might upregulate Klf4 expression to promote SLURP1 release. Another question that needs to be addressed is how SLURP1-α7 nAChR mediated the pathogenesis process of *E. coli* K1 meningitis. Previous studies by our groups and others have reported that nuclear factor-κB (NF-κB) is critical for *E. coli* K1 entry into the CNS (51, 52). Actually, Chernyavsky and coworkers have reported that SLURP1 can bind to α7 nAChR, activate the Raf-1/MEK1/ERK1/2 cascade to modulate NF-κB signaling (53). It has been demonstrated in our previous research that NF-κB modulation, CaMKII, ERK, and protein kinase C are involved in α7 nAChR-mediated signaling (7, 52, 54). It is most likely that the same pathway may contribute to SLURP1-mediated signaling as SLURP1 is an endogenous α7 nAChR ligand. It thus seems that SLPRP1-α7 nAChR-NF-κB cascade might be critical for *E. coli* K1 meningitis.

The limitation of the present study is that the recombinant SLURP1 used was not a native one. Recently, several studies reported the contradictory role of SLURP1 on α7 nAChR based on recombinant SLURP1 with N- and/or C-terminal extensions. In 2003 (22), it was demonstrated that a recombinant SLURP1 containing N-terminal hemagglutinin tag and C-terminal myc tag, could potentiate the α7 nAChRs-mediated responses, while a recent study by Lyukmanova et al. (38, 46) has reported the inhibitory role of recombinant SLURP1, which only added a Met residue in its N-terminal. However, this inhibitory effect was not observed in the case of a synthetic human SLURP1, which is identical with the amino acid sequence of the native source (55). These contradictory findings indicate that additional extensions may produce marked changes in the functional activity of SLURP1. To the best of our knowledge, we believe that SLURP1 may act as a positive modulator, because mutations in SLURP1 cause Mal de Meleda (an inflammatory palmoplantar hyperkeratosis), and α7 nAChR plays a central role in the differentiation of stratified squamous epithelium (22, 56, 57). In spite of this, the conclusion that SLURP1 facilitates *E. coli* K1 crossing the blood-brain barrier needed to be further verified by using the native SLURP1.

Taken together, the present study reveals that SLURP1, an endogenous α7nAChR ligand, is the key mediator for *E. coli* K1 meningitis pathogenesis. Blocking initial SLURP1-α7nAChR interaction would be an attractive strategy for preventing *E. coli* K1 meningitis.

## Data Availability Statement

The original contributions presented in the study are included in the article. Further inquiries can be directed to the corresponding authors.

## Ethics Statement

The animal study was reviewed and approved by Medical Ethics Committee of Southern Medical University.

## Author Contributions

HC, SH, TZ, and XH conceived and designed the experiment. XH, LW, FC, LQL, JG, YW, TH, and ZG performed the experiment. ZG, LW, XH, LL, PZ, TZ, JG, and FC analyzed the data. SH and PZ contributed reagents/materials/analysis tools. XH, HC, JG, BL, TH, and SH participated in its design and coordination and helped in drafting the manuscript. All authors contributed to the article and approved the submitted version.

## Funding

This work was financially supported by the National Natural Science Foundation of China (No. 81871198; 81801985; 81873762; 82060192), China Postdoctoral Science Foundation (No. 2020M682808; 2021T140299), the Key Research Program of Hunan Provincial Department of Science and Technology, China (No. 2022SK2032), Postdoctoral innovative practice project of Jiang Meng (No. JMBSH2020B04), and the Yunnan Key Laboratory of Children’s Major Disease Research (202005AG070073).

## Conflict of Interest

The authors declare that the research was conducted in the absence of any commercial or financial relationships that could be construed as a potential conflict of interest.

## Publisher’s Note

All claims expressed in this article are solely those of the authors and do not necessarily represent those of their affiliated organizations, or those of the publisher, the editors and the reviewers. Any product that may be evaluated in this article, or claim that may be made by its manufacturer, is not guaranteed or endorsed by the publisher.
