# Supplementary figures and images for: Endogenous α7 nAChR Agonist SLURP1 Facilitates Escherichia coli K1 Crossing the Blood-Brain Barrier
Source: Front Immunol. 2021 Oct 14;12:745854. doi: 10.3389/fimmu.2021.745854 (PMC8552013; doi:10.3389/fimmu.2021.745854)

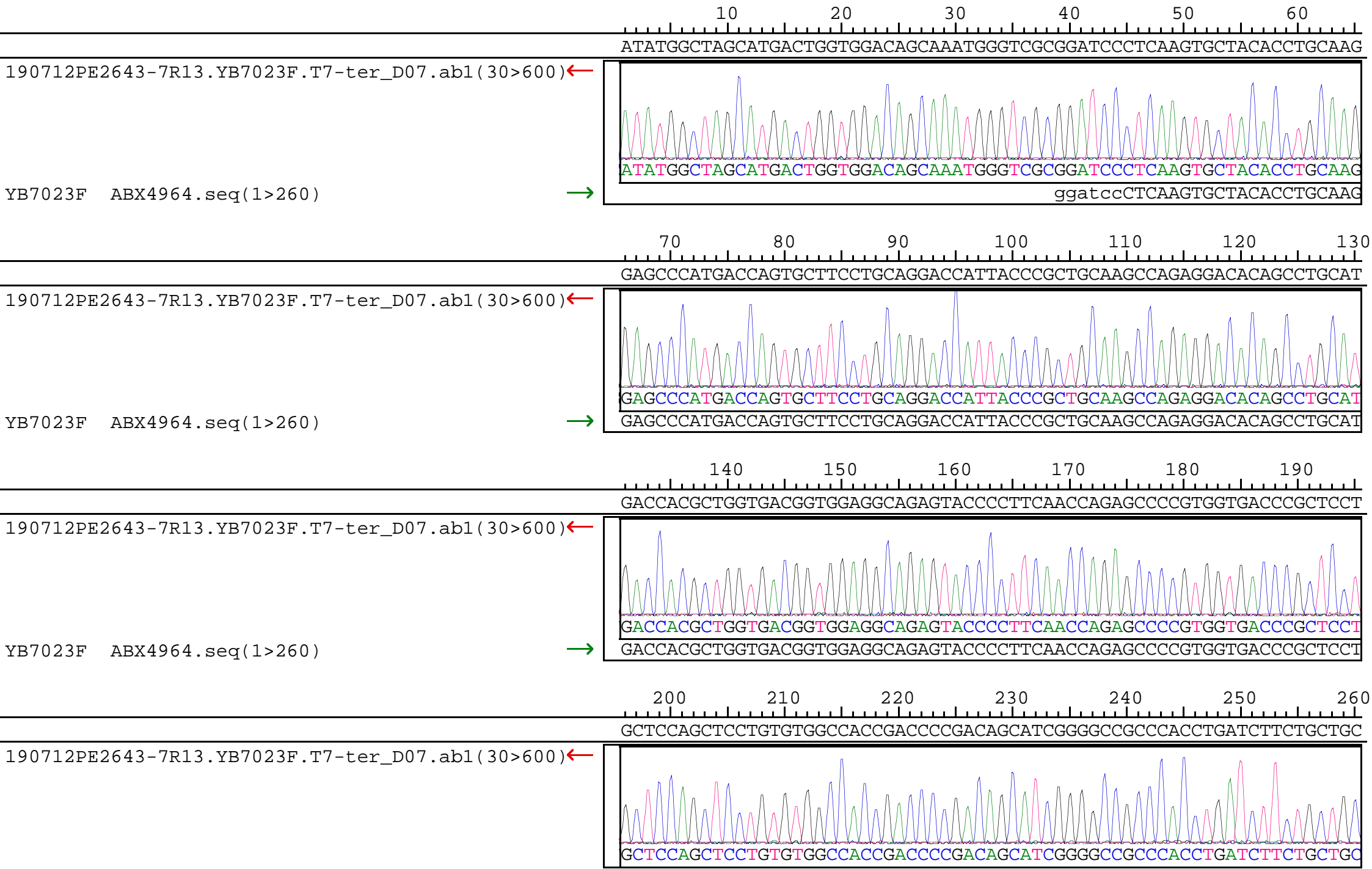

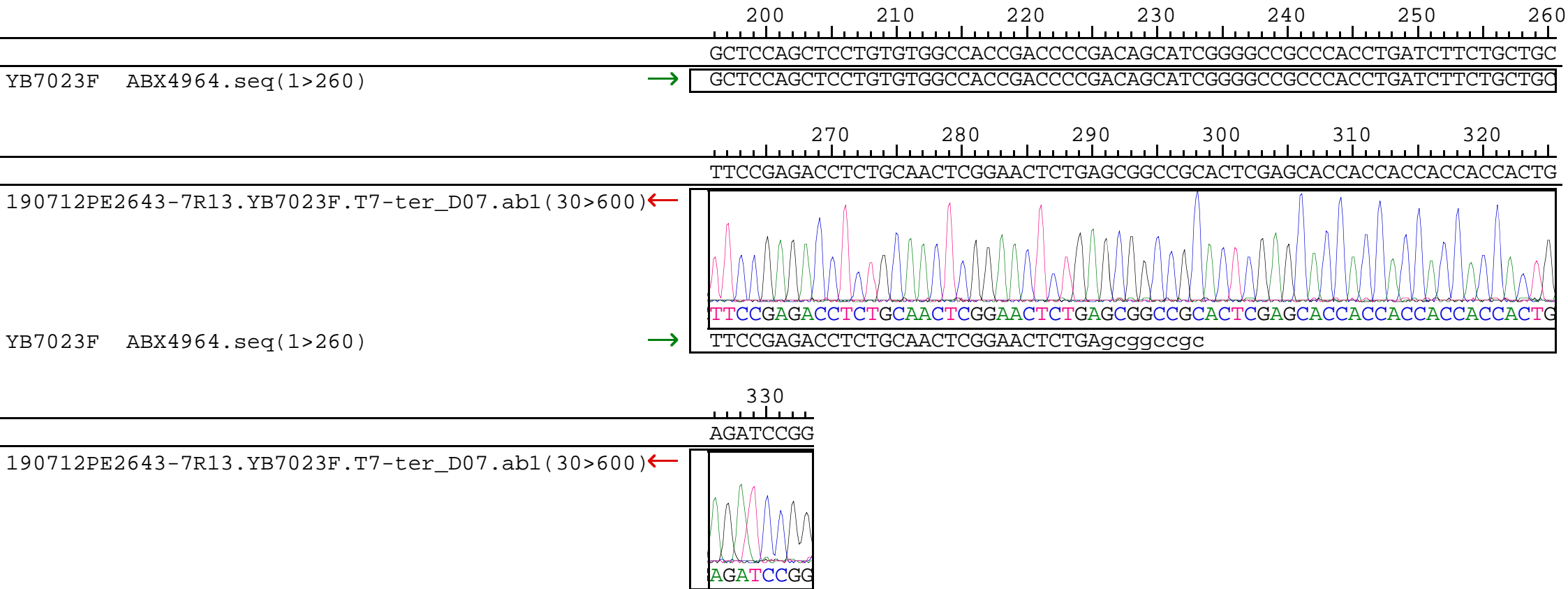

Supplement: Supplementary file 1 [file DataSheet_1.pdf]
