# Supplementary material for: Endogenous α7 nAChR Agonist SLURP1 Facilitates Escherichia coli K1 Crossing the Blood-Brain Barrier
Source: Front Immunol. 2021 Oct 14;12:745854. doi: 10.3389/fimmu.2021.745854 (PMC8552013; doi:10.3389/fimmu.2021.745854)

Figure S1. The amino acid sequence, double-digestion analysis, protein purity and immunoblot detection of recombinant SLURP1. (**A**) The amino acid sequence of recombinant SLURP1 used in this study. (**B**) Restriction enzyme analysis of the pET-28a-SLURP1. M: DNA marker. Recombinant vector digested by BamH I and Not I releasing the expected fragment size of ~240 bp. (**C**) Coomassie brilliant blue staining analysis of the recombination SLURP1 in ~10 KDa size. Lane 1: Prestained protein marker. Lane 2: whole protein lysate of induced bacteria transformed with pET-28a-SLURP1. Lane 5-8: eluted SLURP1 from nickel resin. Right panel, SLURP1 sample from Lane 8 was immunoblot detected using anti-SLURP1 antibody.

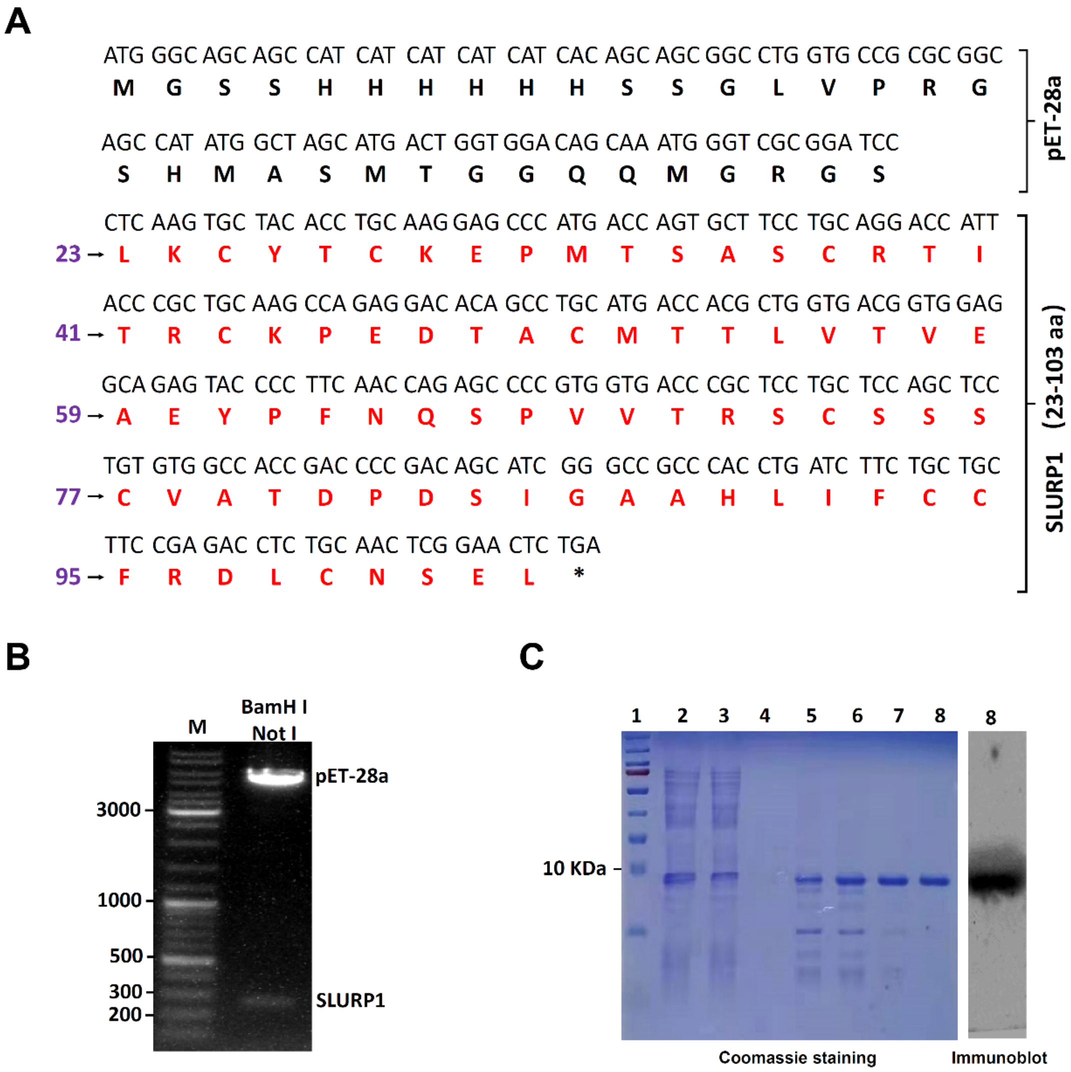

Supplement: Supplementary file 2 [file DataSheet_2.pdf]
